# Supplementary figures and images for: Natural language processing for cognitive therapy: Extracting schemas from thought records
Source: PLoS One. 2021 Oct 18;16(10):e0257832. doi: 10.1371/journal.pone.0257832 (PMC8523074; doi:10.1371/journal.pone.0257832)

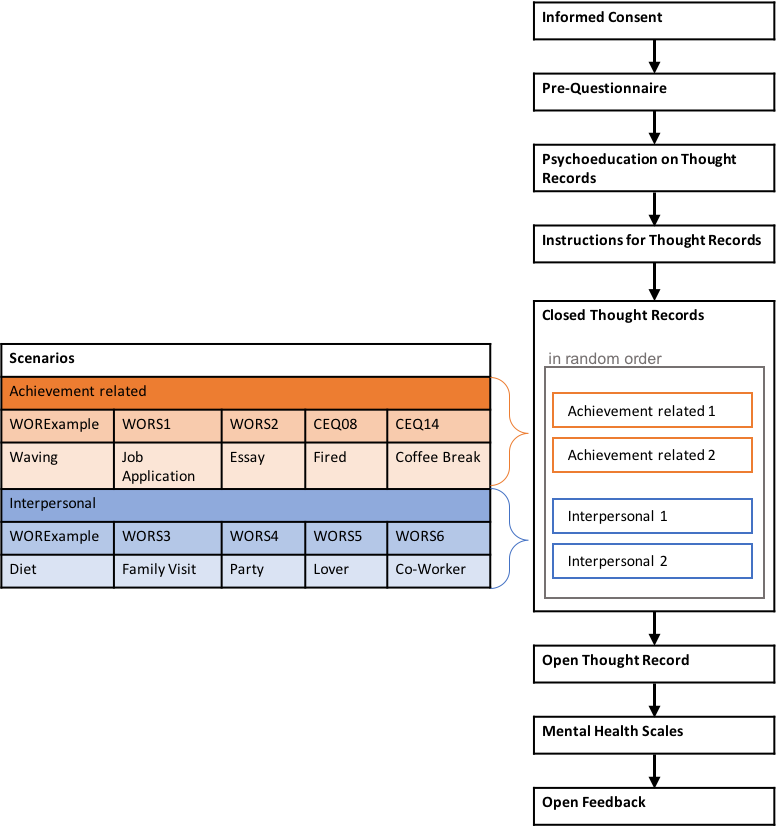

Supplement: S1 Appendix — Figure displays the different stages of the experiment as traversed by the participants. (PNG) [file pone.0257832.s001.png]

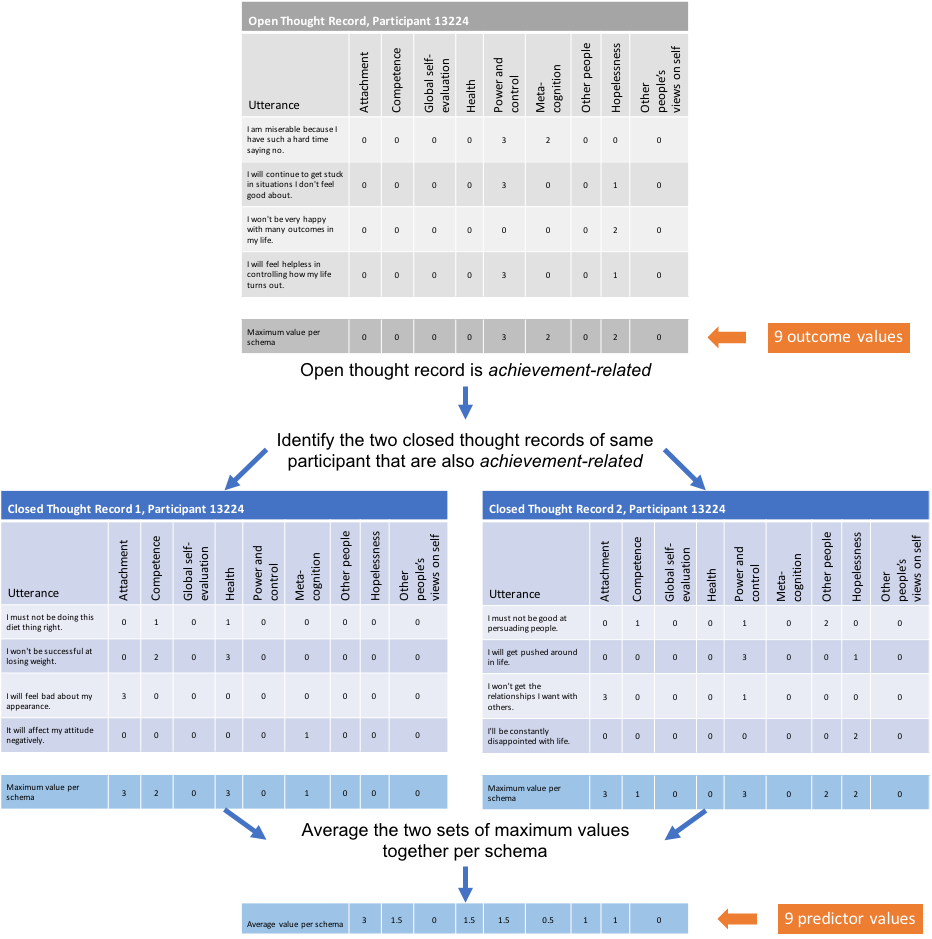

Supplement: S2 Appendix — Graphical illustration of how we determined the predictor and outcome variables for the nine models of hypothesis 3. (PNG) [file pone.0257832.s002.png]
