# Supplementary material for: Natural language processing for cognitive therapy: Extracting schemas from thought records
Source: PLoS One. 2021 Oct 18;16(10):e0257832. doi: 10.1371/journal.pone.0257832 (PMC8523074; doi:10.1371/journal.pone.0257832)
Supplement: S3 Appendix — Table that summarizes the main outcomes of the five linear models that were fit to assess whether there is a link between schemas and outcomes on various mental health questionnaires. (PDF) [file pone.0257832.s003.pdf]

Table that summarizes the main outcomes of the five linear models that were fit to assess whether there is a link between schemas and outcomes on various mental health questionnaires.

**Schema**	**HDAS-D**				**HDAS-A**				**BDI-IA**				**CD-R**				**CD-A**			
	b	β	t	*p*	b	β	t	*p*	b	β	t	*p*	b	β	t	*p*	b	β	t	*p*
Attachment	0.06	0.01	0.24	0.81	-0.13	-0.03	-0.51	0.61	0.29	0.03	0.49	0.63	-0.13	-0.01	-0.22	0.83	-0.39	-0.03	-0.58	0.56
Competence	-0.24	-0.05	-0.90	0.37	-0.24	-0.05	-0.85	0.40	-0.85	-0.07	-1.25	0.21	0.11	0.01	0.16	0.87	0.82	0.06	1.07	0.28
Global self-evaluation	0.31	0.09	1.56	0.12	0.63	0.18	2.98	**<0.01**	1.25	0.15	2.49	0.013	2.11	0.24	4.08	**<0.001**	2.06	0.21	3.64	**<0.001**
Health	0.69	0.10	1.59	0.11	0.57	0.07	1.26	0.21	1.90	0.10	1.75	0.08	1.00	0.05	0.89	0.37	1.52	0.07	1.25	0.21
Power and Control	0.92	0.11	1.86	0.06	0.94	0.10	1.80	0.07	1.39	0.06	1.12	0.27	3.44	0.15	2.69	**<0.01**	2.31	0.09	1.66	0.10
Meta-cognition	0.90	0.04	0.77	0.44	1.36	0.06	1.12	0.27	1.80	0.03	0.62	0.54	-2.22	-0.04	-0.74	0.46	-0.65	-0.01	-0.20	0.84
Other people	-0.24	-0.01	-0.21	0.83	-0.80	-0.04	-0.67	0.50	0.56	0.01	0.19	0.85	-2.24	-0.04	-0.76	0.45	-2.96	-0.05	-0.92	0.36
Hopeless	-0.06	-0.01	-0.11	0.92	0.15	0.01	0.24	0.81	0.68	0.03	0.44	0.66	1.37	0.05	0.87	0.39	1.88	0.06	1.09	0.28
Other's views on self	-0.25	-0.05	-0.84	0.40	0.05	0.01	0.16	0.87	-1.36	-0.10	-1.82	0.07	0.54	0.04	0.71	0.48	-0.01	-0.00	-0.01	0.99

Abbreviations: HDAS-D – Hospital Depression and Anxiety Scale - Depression, HDAS-A – Hospital Depression and Anxiety Scale - Anxiety, BDI-IA – Beck Depression Inventory - I amended, CD-R – Cognitive Distortions - Relatedness, CD-A – Cognitive Distortions - Achievement, b – estimates for the regression coefficient, β – standardized estimates for the regression coefficient
