# Supplementary material for: Natural language processing for cognitive therapy: Extracting schemas from thought records
Source: PLoS One. 2021 Oct 18;16(10):e0257832. doi: 10.1371/journal.pone.0257832 (PMC8523074; doi:10.1371/journal.pone.0257832)
Supplement: Attachment — Submitted filename: review.docx [file pone.0257832.s004.docx]

Paper:

Natural language processing for cognitive therapy: extracting schemas from thought records

Review:

The paper demonstrates an NLP based psychotherapy approach that attempts to automatically detect underlying maladaptive schemas from the thought record forms. For demonstrating that such approach would work, the paper proposes 4 hypothesis H1, H2, H3 and H4 and conducts experiments towards each.

Strength of paper:

The paper explains the methods clearly and contributes a dataset of completed thought records that was collected through the online crowdsourcing platform. Paper discusses the results that supports H1 and partially for H3, H4.

Possible improvements:

Line [265]

The post-questionnaire comprised three items asking participants how difficult and how enjoyable they found it to complete a thought record, and to indicate how many thought records they think they would complete if they were asked to complete a thought record daily for a period of seven days.

How would you ensure that participants are not influenced by secondary motivation of “earning more money” rather than truthfully answering as they are not actual patients but are paid for recording the thoughts. In short, how did you nullify or minimize the inherent monetary motivation for completing the tasks that these participants have but which actual patients won’t.

Table 4

Explain why you get nan as value for Spearman correlation. For Meta-cognition schema, the bes corelation is 0.11 which is pretty weak. Explanation of why possibly you got consistently weak correlation across models for this schema, Power and Control and Other People schema.

Abstract mentions that a Pretrained natural language processing software was used. It would be better to clarify whether this nlp software was deep learning based and whether it is pretrained on common text corpus or corpus specific to psychotherapy.
