# Supplementary material for: Natural language processing for cognitive therapy: Extracting schemas from thought records
Source: PLoS One. 2021 Oct 18;16(10):e0257832. doi: 10.1371/journal.pone.0257832 (PMC8523074; doi:10.1371/journal.pone.0257832)
Supplement: Attachment — Submitted filename: Response_to_Reviewers_Round2.docx [file pone.0257832.s006.docx]

Dear Editor,

Thank you for forwarding the second round of reviews to us. We would also like to thank the reviewers for their second evaluation of the manuscript and for pointing out some remaining points-of-improvement.

We have addressed the two points raised by Reviewer #2. Please find below a brief description of how we done this.

We have also submitted the edited manuscript with tracked changes (only the new changes).

## Reviewer #1 - No additional points raised

Reviewer #2

1. In Table 4 it ought to be made clear what "nan" is and the reasons for it. My assumption is nonconvergence, but the authors should discuss it.

This point had previously also been raised by the first reviewer, and we had addressed it in the text. However, we regard the fact that Reviewer #2 raised it again as an indication that it is still not sufficiently clear. In the table notes, we have now included a short comment stating the meaning of the abbreviation, explaining why the algorithm returns nan-values, and referring to the text for more details [see table notes under Table 4 and lines 565-573 for the in-text details, with the latter added after the previous reviewing round].

2. Does the fact that the authors' schema for the most part fail to predict depression and anxiety outcomes challenge the validity of the methodology? After all, clinicians primarily care about schema because of their place in the theory underlying cognitive behavioral therapy. I do think that the authors should be more thoughtful in their discussion of this finding.

This is an interesting point raised by the reviewer. As the reviewer already figured correctly in the previous reviewing round, we are no psychologists. Our primary interest in testing this hypothesis was not of clinical nature but rather to be able to possibly improve the algorithm. Nonetheless, we do not think that the fact that no link was found challenges or invalidates the methodology. This is because the methodology is based on thought records, which are momentary assessments of a person’s thoughts, while the way in which depression and anxiety outcomes are assessed is with inventories that are symptom-based and longer term. However, if the methodology is valid (and we believe it to be), we would certainly expect to see such links showing in a clinical population that regularly completes thought records over an extended period (as is also done in therapy) and holds schemas which the algorithm can predict relatively reliably (e.g., Attachment or Competence schemas). We have added a few sentences to express this in the discussion [see lines 704-710].
